# Identification of self-incompatibility alleles in Quince (*Cydonia oblonga* Mill.)

**DOI:** 10.1371/journal.pone.0297595

**Published:** 2024-02-08

**Authors:** Sara Sadeghnejad, Hamid Abdollahi, Daryoush Davoodi, Maryam Tatari, Mahmoud Khosroshahli

**Affiliations:** 1 Department of Plant Breeding and Biotechnology, Science and Research Branch, Islamic Azad University, Tehran, Iran; 2 Temperate Fruits Research Center, Horticultural Sciences Research Institute, Agricultural Research, Education and Extension Organization (AREEO), Karaj, Iran; 3 Department of Nanotechnology, Agricultural Biotechnology Research Institute of Iran (ABRII), Karaj, Iran; 4 Horticulture Crops Research Department, Isfahan Agricultural and Natural Resources Research and Education Center, Agricultural Research, Education and Extension organization (AREEO), Isfahan, Iran; University of Delhi, INDIA

## Abstract

The Quince (*Cydonia oblonga* Mill.), typically known for its self-compatibility, surprisingly presents a degree of self-incompatibility. This research focused on exploring the diversity within the self-incompatibility gene locus (S) in various *C*. *oblonga* genotypes. Through meticulous DNA sequencing, the study sought to unearth potential novel S alleles. In the process of genotyping the S gene across multiple quince genotypes, not only were the previously documented S1 and S2 alleles identified, but this investigation also uncovered two previously unrecognized alleles, termed S4 and S5. These alleles, particularly S4, emerged as the most prevalent among the tested genotypes. To corroborate the findings derived from DNA sequencing, the study employed pollen tube growth germination assays. These assays elucidated a higher pollen germination rate in the Ardabil2 genotype in contrast to Behta. Additionally, the study involved assessing pollen tube growth in both Ardabil2 and Behta through cross-pollination techniques, meticulously tracking the development of pollen tubes at various stages. Remarkably, the outcomes demonstrated that the Behta genotype possesses self-incompatibility, whereas the Ardabil2 genotype showcases a notable degree of self-compatibility. This groundbreaking discovery of new S alleles in quince not only affirms the species’ self-compatibility but also sheds light on the complexities of allelic diversity and its impact on self-incompatibility. Such insights are invaluable for enhancing the yield of quince orchards through strategic breeding programs.

## Introduction

Quince (*Cydonia oblonga* Mill., 2n = 2x = 34), a member of the Rosaceae family, is predominantly found in temperate regions globally, tracing its origins to Western Asia, notably in the northern regions of Iran, Turkmenistan, the Caucasus, Armenia, Azerbaijan, and the Russian Federation [1, 2]. Ranking as the third most economically significant fruit tree in the pome fruit subgroup, following apples and pears [3], quince is celebrated for its health-enhancing attributes, attributed to its robust antioxidant activities and rich phenolic content [4].

The species has witnessed an upsurge in cultivation, particularly in Iran, showcasing a burgeoning popularity [2] and a wealth of genetic diversity and superior fruit quality within Iranian cultivars [5].

Quince orchards, however, confront significant challenges, notably the premature loss of flowers and fruits during early growth stages. This issue is multifaceted, influenced by factors such as water availability, soil quality, pruning practices, nutritional factors, and varying environmental conditions affecting pre- and post-pollination [6, 7]. The interplay between pollination and compatibility is crucial in determining fruit production. A notable gap in pollination and an incomplete understanding of the mechanisms behind self-compatibility and self-incompatibility have been identified as primary contributors to the suboptimal fruit set in quince [7]. Despite reports of self-compatibility, a prevalence of self-incompatibility in a majority of quince cultivars and genotypes has been observed, suggesting a need for its consideration in breeding programs aimed at developing cultivars with enhanced fruit set and yield [7].

Extensive research has been dedicated to understanding the genetic architecture of self-incompatibility in the Rosaceae family, including quince [8–11]. This family is characterized by a gametophytic self-incompatibility system, governed by a specific gene locus (S-locus) and encompassing a broad spectrum of self-incompatibility alleles (S-allele) [10]. In this system, when a pollen grain’s S allele matches one of the pistil’s S alleles, it triggers a mechanism that limits fertility and fruiting by inhibiting self-pollen tube germination and promoting cross-pollination, thereby precluding the production of homozygous individuals [12]. The S-RNase gene in the style is a key component in governing this self-incompatibility [13].

Initial discovery of the S1 allele (S1, MF281258) in quince was reported by Talaei et al. [14] revealing its significant similarity with alleles in related species such as *Malus domestica* Borkh and *Pyrus communis* L. This was followed by further research led by Lev-Mirom, Y., and Goldway, unveiling additional alleles, namely S2 (MH937559) and S3 (MH937560), further enriching the genetic landscape of quince.

Tatari et al. [7] conducted a study at the Horticultural Research Station of Isfahan, Iran, which is recognized as a vital collection of quince varieties worldwide. Their aim was to identify the most suitable pollinizer for different quince genotypes. However, the language used in the article could benefit from some improvement in expression. Various techniques can be employed to investigate the compatibility or incompatibility between different cultivars and identify appropriate pollinators. These methods encompass controlled pollination, observation of pollen tube growth using a fluorescence microscope, extraction of ribonuclease from the style, S allele-specific PCR, and nucleotide sequencing related to incompatibility [15]. In this particular study, the researchers utilized the S allele-specific PCR method in conjunction with the observation of pollen tube growth. The identification and determination of self-incompatibility alleles in a variety or genotype, as well as the recognition of genetic structures associated with self-compatibility, are crucial aspects of breeding programs. Consequently, the primary objective of this study was to evaluate self-incompatibility and discover new S alleles in quince genotypes.

## Material and methods

### Plant material

**Fig 1:**
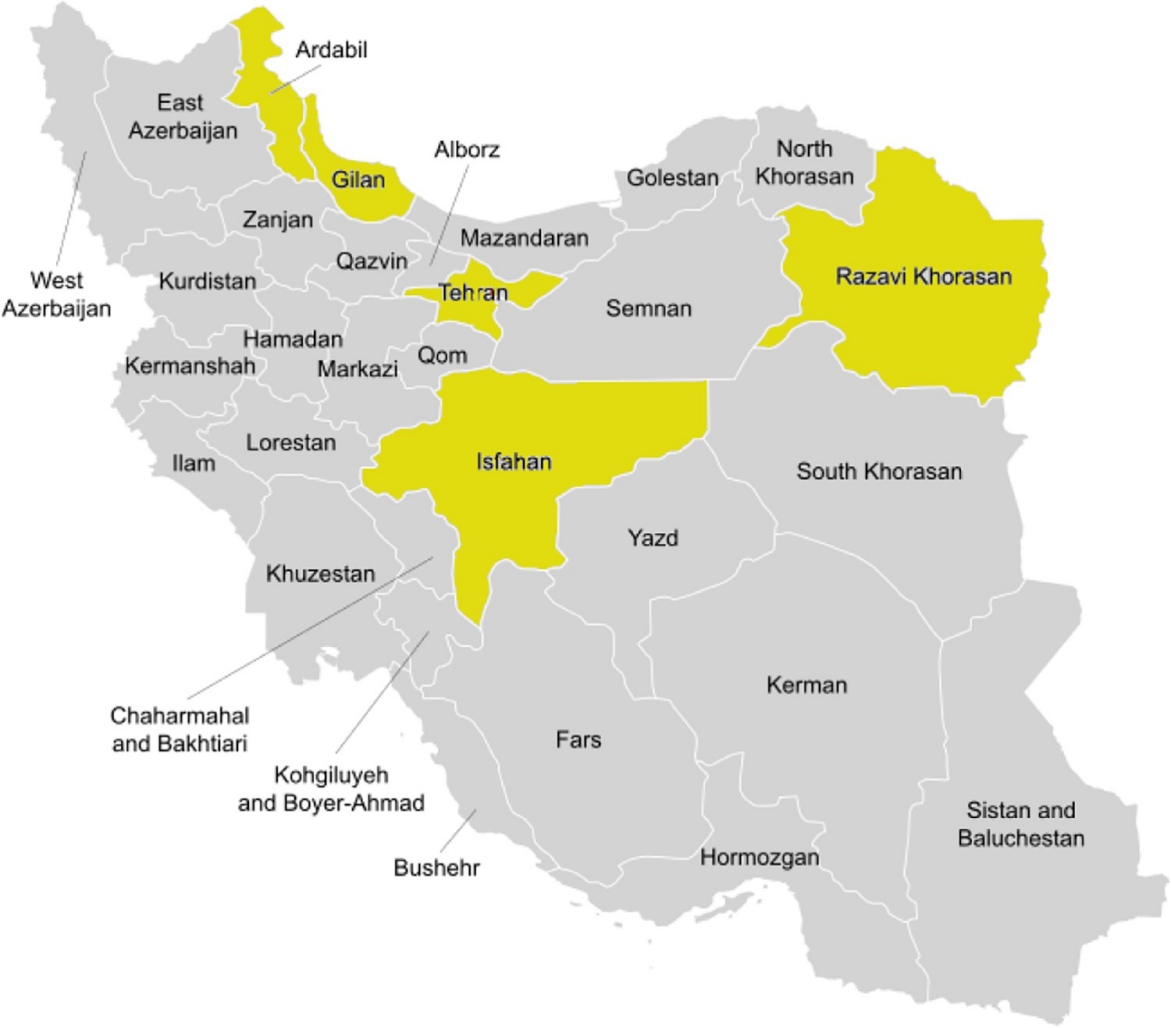
Spread of the quince genotypes in Iran. Underlying map sourced from Ali Zifan, Wikimedia Commons (https://commons.wikimedia.org/wiki/File:Iran_provinces.svg).

In this study, 27 genotypes of quince (Table 1) were obtained from the germplasm collection of the Temperate Fruits Research Center, Horticultural Sciences Research Institute, Agricultural Research, Education and Extension Organization (AREEO), located in Karaj, Iran (35.85°N, 50.86°E, 1245 m height). The necessary permit for access to the field site was obtained from the Horticultural Sciences Research Institute, AREEO. The S gene locus and pollen tube growth were used to determine self-incompatibility in the surveyed quince genotypes. The quince genotypes were collected from five provinces in Iran: Khorasan, Isfahan, Ardabil, Guilan, and Tehran, as shown in Fig 1.

**Table 1:** The origin of the quince genotypes collected from different parts of Iran as a center of origin and center of diversity.

No.	Genotype	Origin	No.	Genotype	Origin
1	Moghavem 1 (MV1)	Khorasan	15	Amroudi Kowsar	Ardabil
2	Moghavem 2 (MV2)	Khorasan	16	Haj Agha Kishi	Ardabil
3	Gardandar	Khorasan	17	Ardabil2	Ardabil
4	Isfahan Oghaf (EO)	Khorasan	18	Ardabil3	Ardabil
5	SVS1	Isfahan	19	Ardabil5	Ardabil
6	NB2	Isfahan	20	Ardabil7	Ardabil
7	PK2	Isfahan	21	Givi Kowsar	Ardabil
8	SVNZ02B01 (SVS2)	Isfahan	22	Sibi Kowsar	Ardabil
9	KM1	Isfahan	23	ANZ02B01 (AS2)	Gilan
10	Vidoja (KVD1)	Isfahan	24	ASM2	Gilan
11	KVD2	Isfahan	25	ASP1	Gilan
12	Behta (PH2)	Isfahan	26	NM	Gilan
13	KVD4	Isfahan	27	LA3	Tehran
**14**	**ET1**	**Isfahan**			

Some genotypes were selected from Ardabil province, as a key center of origin, and others were collected from other provinces of Iran as centers of diversity.

### Genotyping experiment

To extract genomic DNA from the 27 quince genotypes, young and healthy leaves were used and the CTAB method was employed [16]. The extracted DNAs were evaluated for quality and quantity using 1% agarose gel electrophoresis and nanodrop at 260 and 280 nm wavelengths, respectively. DNA was diluted with water to a concentration of 20 ng/μl.

Genes that perform similar functions or belong to the same gene family often share conserved regions. In cases where gene sequences are unavailable for a particular plant species, designing primers based on these conserved regions from known genes can facilitate the amplification of the desired DNA fragments via PCR [17, 18]. Due to the limited sequencing of self-incompatibility (SI) genes across the plant species, primers from SI genes in pear were utilized as forward and reverse primers to amplify SI genes in quince. The specific primers used were sall [19] and FB [20], as indicated in Table 2.

**Table 2:** Nucleotide sequences of the primers used in this research.

Primers	Nucleotide sequences	References	Species
Sall-F	TTTACGCAGCAATATCAGC	Mota *et al*., 2007	*Malus*
**FB-R1**	GCATTTTCAATATCCACCAG	Babaei *et al*., 2012	*Pyrus*

Polymerase chain reaction (PCR) was performed on the DNA of quince cultivars using a BIO RAD T100™ thermocycler. The used primer pair was diluted with deionized distilled water at a concentration of 10 μM based on the provided OD of the primer. PCR components include 12.5μl PCR master (Taq 2x Master red, 1.5 mM), 1μl of each forward and reverse primers, 3 μl DNA, and 7.5 μl of deionized distilled water for each 25μl reaction. The PCR amplification was carried out using a program consisting of an initial denaturation step at 94°C for 3 minutes, followed by 35 cycles of denaturation at 94°C for 45 seconds, annealing at 50°C for 1 minute, and extension at 72°C for 1 minute. The final extension step was performed at 72°C for 10 minutes. The 100 bp DNA Ladder plus (CinaGen Co.) with Cat. No. SL7041 (PR911653) was used. To visualize and analyze the resulting bands, we used a horizontal electrophoresis device with 1.2% agarose gel, and the bands were separated with a voltage of 110 volts. Subsequently, the PCR products were sequenced using the Sanger sequencing method by Niagenenoor Corporation (Niagenenoor, Tehran, Iran).

### Bioinformatics and phylogeny analysis

In this study, the phylogenetic analysis of all self-incompatibility alleles from three seed plants, namely apple, quince, pear, and common medlar was conducted utilizing available sequence data sourced from the NCBI database. The obtained sequences, acquired through the process of sequencing, underwent editing procedures employing Chromas software. Subsequently, the comparison of these sequences with distinct alleles was carried out employing BLAST software to assess their similarity. The construction of the phylogenetic tree was accomplished using the neighbor-joining method, and the resultant tree was visualized using MEGA11 software [21].

### Pollen germination and pollen tube growth

To evaluate the self-incompatibility status of the Ardabil2 and Behta genotypes, pollen tube growth was assessed using fluorescence microscopy. From each genotype, branches with flower buds were cut and placed in a 4% sucrose solution at room temperature. The anthers of opened flowers were separated and left on paper at room temperature for 24 to 48 hours to dry the pollen. The pollen grains were then transferred to sterile vials and stored at 4°C in the refrigerator until pollination.

To ensure the viability of the pollen grains, they were distributed in a petri dish containing a medium supplemented with 1% agar, 15% sucrose, and 50 mg/L of boric acid, as described by Dalkilic and Mestav [22]. The germination percentage of the pollen grains was recorded using a light microscope after 24 hours under laboratory temperature. Branches were collected from individual genotypes and transported to the laboratory for further analysis. Pollen grains were carefully extracted from each branch and placed into separate sterile vials. Subsequently, a portion of the pollen grains from each vial was cultivated to assess their viability and germination potential. Pollen samples exhibiting favorable characteristics, such as viability and germination rates exceeding 75%, along with successful pollen tube growth, were selected for subsequent experiments. These chosen samples served as the primary source of pollen grains or the main reserve for further investigations.

To perform artificial pollination, appropriate and uniform branches containing flower buds at the swollen-bud stage were randomly selected in the four geographical directions of the target trees. The flowers at the balloon stage were emasculated, and all other flowers were removed from the branches, which were then covered with cloth bags. After the stigma became receptive to accept pollen grains, the emasculated flowers were pollinated with the selected pollen using a glass rod for each cross. The pollinated flowers were then covered with cloth bags and labeled.

To analyze the growth of pollen tubes, 10 pistils from the pollinated flowers were collected at 24, 72, and 120 hours after controlled pollination and fixed in FAA fixative solution (5% formaldehyde, 5% glacial acetic acid, 90% ethanol 70%). After five months, the samples were removed from the fixative solution, washed three times with distilled water, and then placed in 8 N NaOH for 24 hours. The samples were then washed three times with distilled water and placed in a 0.1 N potassium phosphate (K_3_PO_4_) solution containing 1% aniline blue dye for 3–4 hours. The ovary was separated from the style under sterile conditions, and the growth of the pollen tube was examined in the style using a fluorescence microscope (Model: Nikon E800 Epi).

The average pollen grain germination on the surface of the stigma and pollen tube penetration were recorded at 24, 72, and 120 hours, in the upper, middle, and end of the style, and compared between different times and different genotypes.

## Results

### S-genotyping and characterization of new alleles

**Fig 2:**
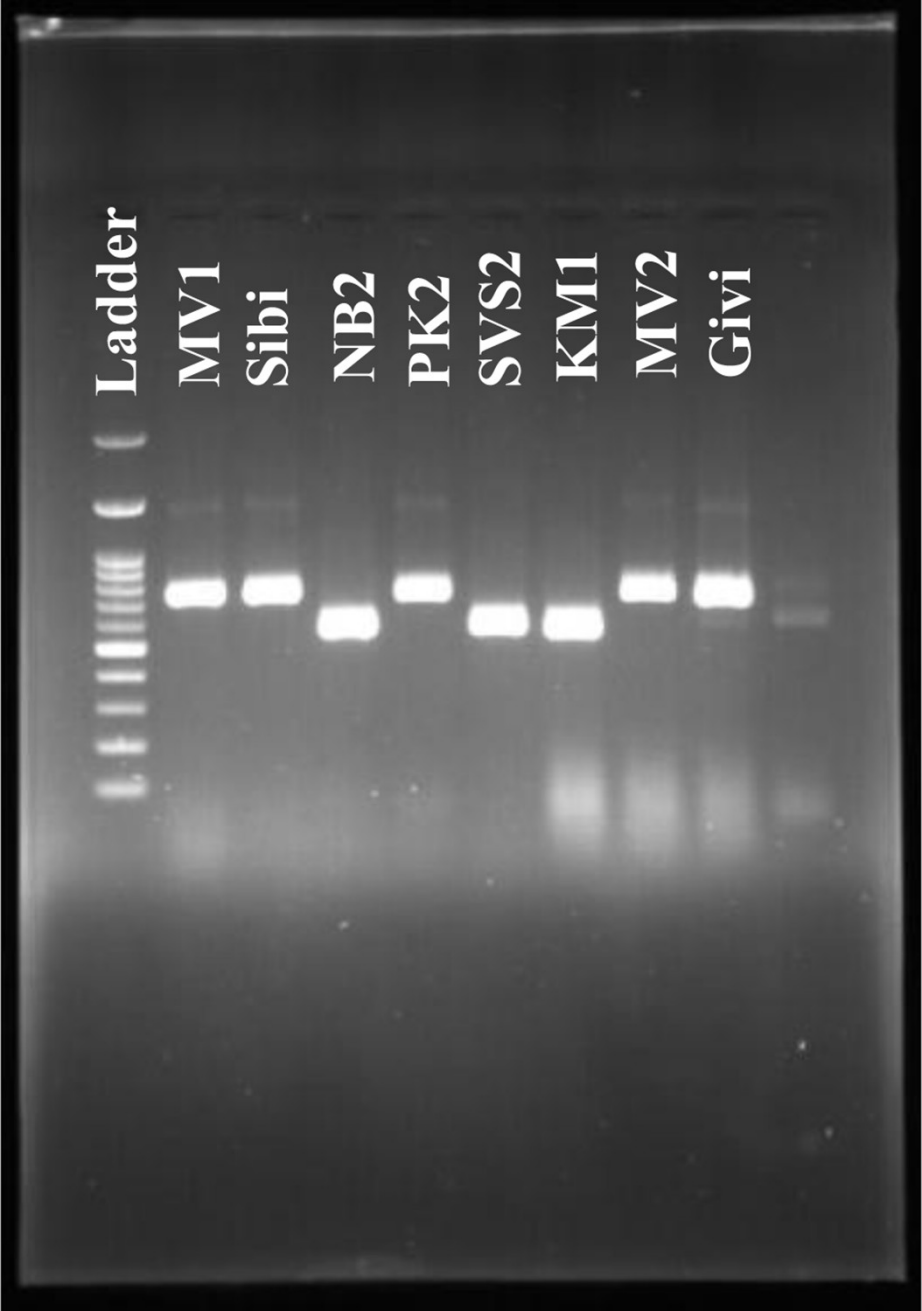
Gel electrophoresis for the quince genotypes.

Fig 2 illustrates the gel electrophoresis outcomes for various quince genotypes, highlighting the genetic diversity using selected primers (Fig 2 and S1 Fig). The amplified DNA fragments, varying from 550 to 750 nucleotides in length among the genotypes, were sequenced to identify the S alleles in quince. DNA sequencing with specific primers revealed the presence of S1, S2, S4, and S5 alleles in the tested genotypes. Genotypes NB2, SVS2, KM1, Vidoja, KVD2, Haj Agha, and Ardabil5 displayed the S1S1 genotype, characterized by a 550-bp nucleotide sequence closely resembling the S1 allele in *C*. *oblonga* (with over 95% similarity). Ardabil7 and LA3 genotypes exhibited a 550-bp sequence akin to the S2 allele in quince, showing 99% similarity. Remarkably, genotypes MV1, MV2, GRD, EO, SVS1, PK2, KVD4, ET1, Amroudi, Ardabil3, Givi, Sibi, AS2, and NM presented a unique 720-bp sequence, not matching any known S alleles in quince. These are thus considered to possess a novel S4 allele, now registered in NCBI GenBank under the accession number MW139301. Additionally, genotypes ASM2, ASP1, and Behta exhibited sequences not matching any known alleles in the Cydonia genus, categorized as a new S5 allele and recorded in GenBank with accession number OP884648 (Table 3). Genotyping of Ardabil2 revealed it possesses both S2 and S4 alleles. Allele frequency analysis (Table 3) showed that the S4 allele was the most prevalent among the studied genotypes, found in 14 genotypes, followed by S1 in 7 genotypes, S5 in 3, and S2 in 2 genotypes. No genotype was found to contain the S3 allele.

**Table 3:** The self-incompatibility related alleles of the quince genotypes.

Quince Genotype	S1	S2	S3	S4	S5	Genotype
MV1	-	-	-	+	-	S4S4
MV2	-	-	-	+	-	S4S4
GRD	-	-	-	+	-	S4S4
EO	-	-	-	+	-	S4S4
SVS1	-	-	-	+	-	S4S4
NB2	+	-	-	-	-	S1S1
PK2	-	-	-	+	-	S4S4
SVS2	+	-	-	-	-	S1S1
KM1	+	-	-	-	-	S1S1
Vidoja	+	-	-	-	-	S1S1
KVD2	+	-	-	-	-	S1S1
KVD4	-	-	-	+	-	S4S4
Behta	-	-	-	-	+	S5S5
ET1	-	-	-	+	-	S4S4
Amroudi	-	-	-	+	-	S4S4
Haj Agha	+	-	-	-	-	S1S1
Ardabil2	+	-	-	+	-	S1S4
Ardabil3	-	-	-	+	-	S4S4
Ardabil5	+	-	-	-	-	S1S1
Ardabil7	-	+	-	-	-	S2S2
Givi	-	-	-	+	-	S4S4
Sibi	-	-	-	+	-	S4S4
AS2	-	-	-	+	-	S4S4
ASM2	-	-	-	-	+	S5S5
ASP1	-	-	-	-	+	S5S5
NM	-	-	-	+	-	S4S4
LA3	-	+	-	-	-	S2S2
Allele frequency	7	2	0	14	3	

**Fig 3:**
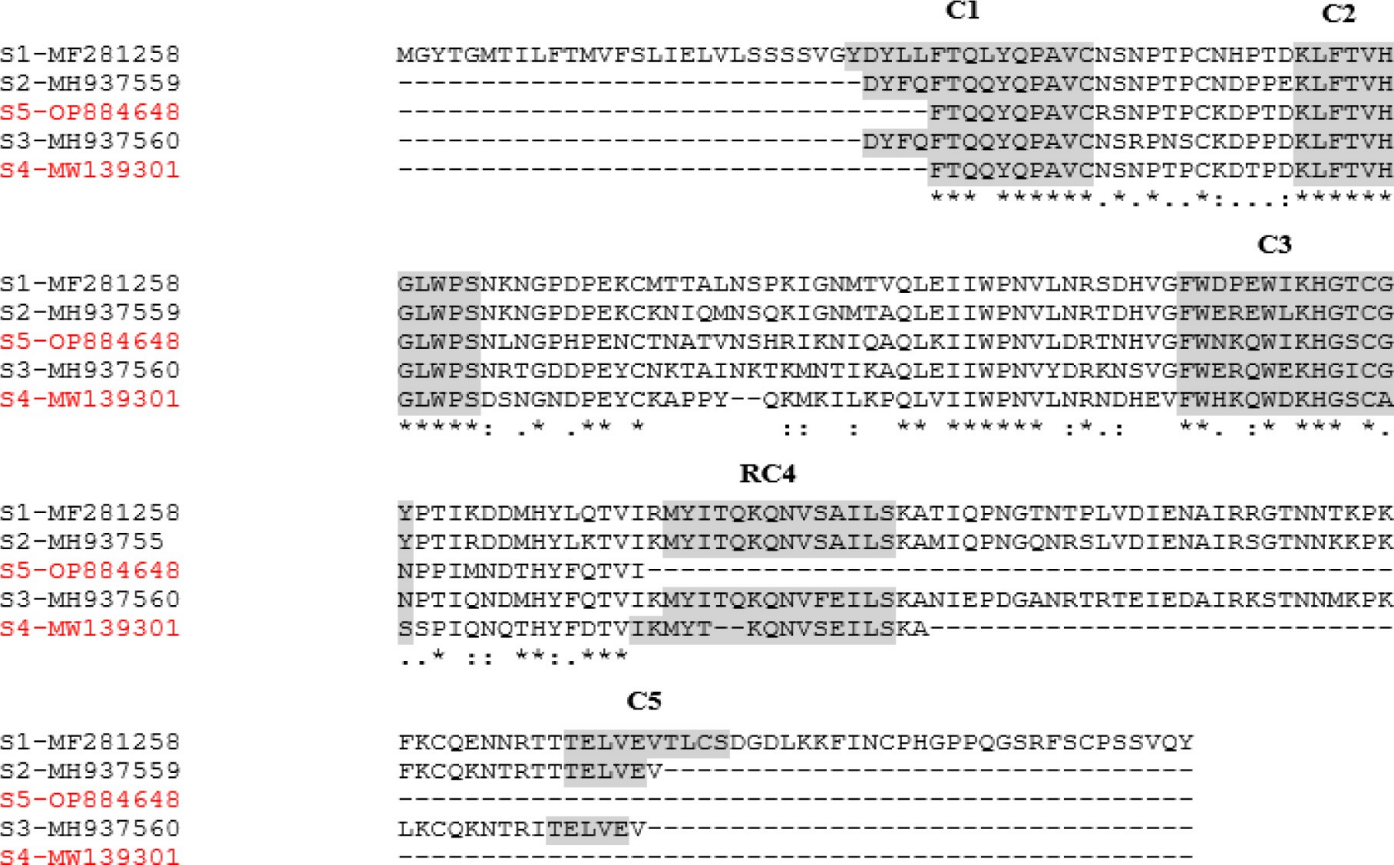
Polypeptide sequence comparison of the five conserved regions of self-incompatibility S1 to S5 alleles in the quince. The conserved motifs of the S alleles were highlighted. The ‘*’ means same sequence in all alleles; the ‘.’ means amino acid difference in all sequences; and the ‘:’ means more abundance of that amino acid.

**Fig 4:**
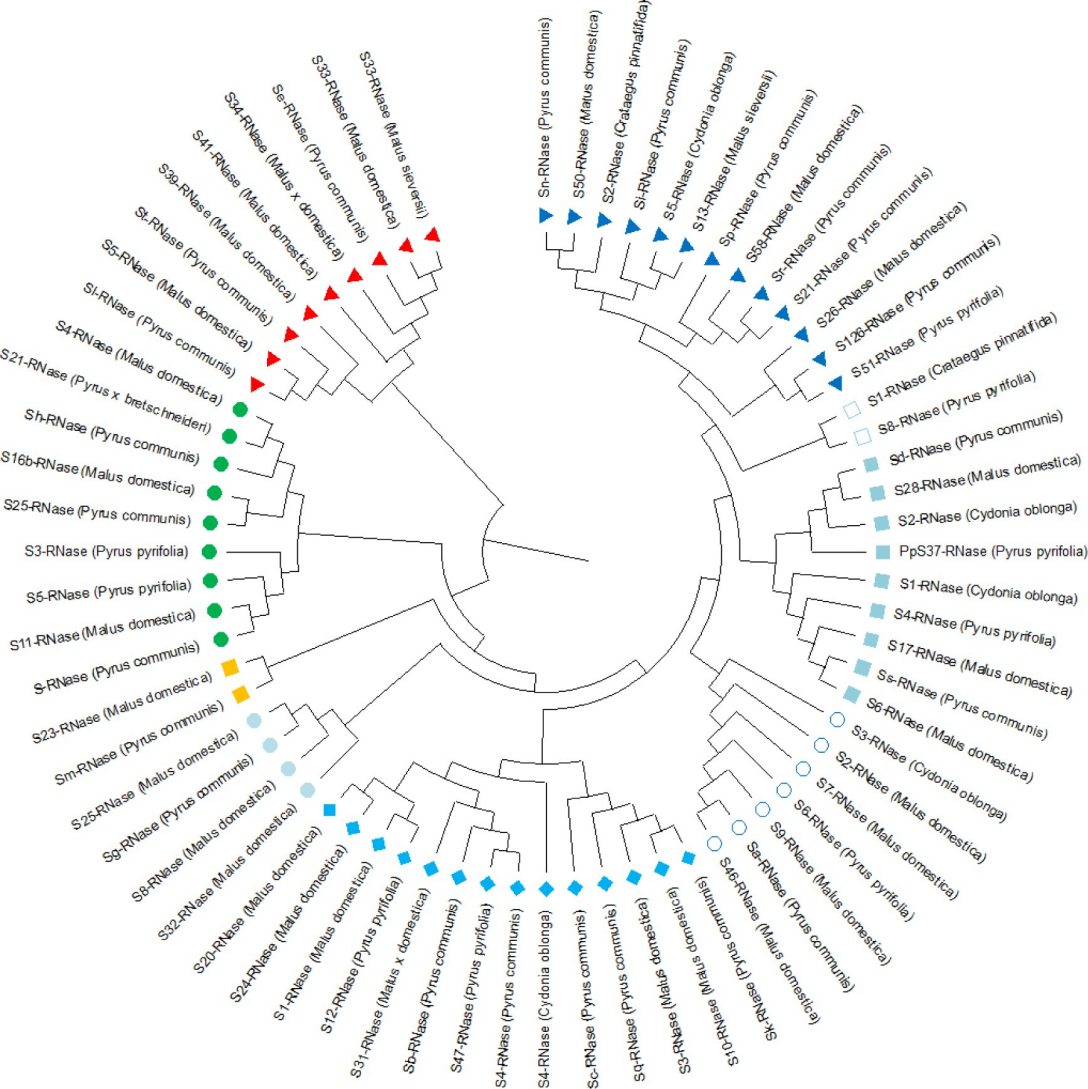
Phylogeny analysis of all self-incompatibility alleles (S-RNase alleles) from apple, quince, pear, and common medlar based on sequence data obtained from the NCBI database.

The aligned nucleotide sequences of quince S alleles demonstrated distinct single nucleotide polymorphisms (SNPs) in the conserved regions of self-incompatibility alleles, namely C1, C2, C3, RC4, and C5, as detailed in Table 4. Notably, the nucleotide diversity identified was corroborated by the amino acid sequences illustrated in Fig 3. Among these regions, C2 showed the highest nucleotide conservation, surpassing C1, while C3, RC4, and C5 exhibited comparatively lower conservation levels, especially in the segments preceding the intron (Table 4). Analysis of the alignment from S1 to S5 alleles in quince revealed the presence of three protein motifs common across all S alleles and two additional motifs found in certain S alleles, as depicted in Fig 3. These findings are in agreement with the similarities observed in DNA sequence alignment. Phylogenetic analysis was conducted on various quince genotypes with differing S alleles, using DNA sequences to explore the relationships between these alleles in quince and in related plant species such as *Pyrus communis*, *Malus domestica*, *Crataegus pinnatifida*, *Malus sieversii*, *Pyrus pyrifolia*, and *Pyrus bretschneideri* (Fig 4). This analysis categorized the genotypes into five subgroups, with *C*. *oblonga* being represented in three of these subgroups. Specifically, the S1 and S2 alleles of quince formed a distinct sub-cluster, whereas the S3, S4, and S5 alleles were segregated into different subgroups. Intriguingly, the phylogenetic study disclosed that while the S1 to S4 alleles of quince bore resemblance to those in related species, the quince S5-RNase allele showed a unique close relation to the S13-RNase allele in *Malus sieversii* (Fig 4).

**Table 4:** Nucleotide sequence comparison of the five conserved regions of self-incompatibility S1 to S5 alleles in the quince genotypes.

Allele	Accession Number	C1 conserved region	C2 conserved region	C3 conserved region	RC4 conserved region	C5 conserved region
S1	MF281258	tacgattatttactatttactcagctatatcagccggccgtatgc	aagttgtttacggttcacggtttgtggccttca	ttctgggacccagagtggatcaaacatggcacctgcgggtat	atgtacataacccagaaacaaaacgtctctgcaatcctctca	actgaattggttgaggtcactctatgcagt
S2	MH937559	gattattttcaatttacgcagcaatatcagccggccgtatgc	aaattgtttacggttcacggtttgtggccttca	ttctgggaaagagagtggctcaaacatggcacctgcgggtat	atgtacataacccagaaacaaaacgtctctgcaattctctca	-
S3	MH937560	gattattttcaatttacgcagcaatatcaaccggctgtctgc	aagttgttcacggttcacggtttgtggccttca	ttctgggaaagacagtgggaaaaacatggcatctgtgggaat	atgtacataacccagaaacaaaacgtctttgaaatcctctca	-
S4	MW139301	-	aagttgtttactgttcacggtttgtggccttca	ttctggcataaacagtgggacaaacatggctcctgtgcgtcg	atcaaaatgtacacaaaacaaaacgtctctgaaatcctctca	-
S5	OP884648	-	aagttgtttacggttcacggtttgtggccttca	ttctggaataaacagtggataaagcatggcagctgtgggaat	–	-
Con.		…gattattttCaATTTACgCAGCaATATCAgCCGGCcGTaTGC	AAgTTGTTtACgGTTCACGGTTTGTGGCCTTCA	TTCTGGgA.a.AcAGTGG. tcAAaCATGGCacCTGtGgGtat	ATgtAcATaacCcagAAACAAAACGTCTcTGaAATcCTCTCA	

### Pollen germination and pollen tube growth efficiency

**Fig 5:**
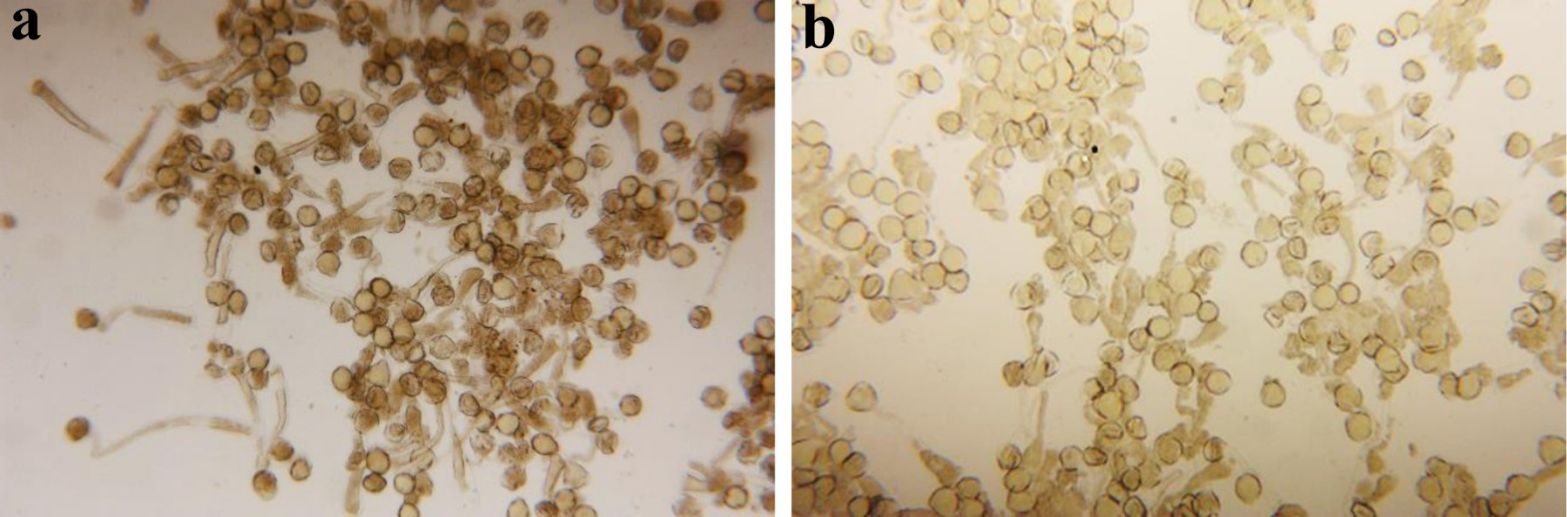
Pollen germination of the Ardabil2 (a) and Behta (b) and genotypes.

The pollen germination of the quince genotypes was assessed under *in vitro* conditions, demonstrating normal germination and growth of pollen grains (Fig 5).

**Fig 6:**
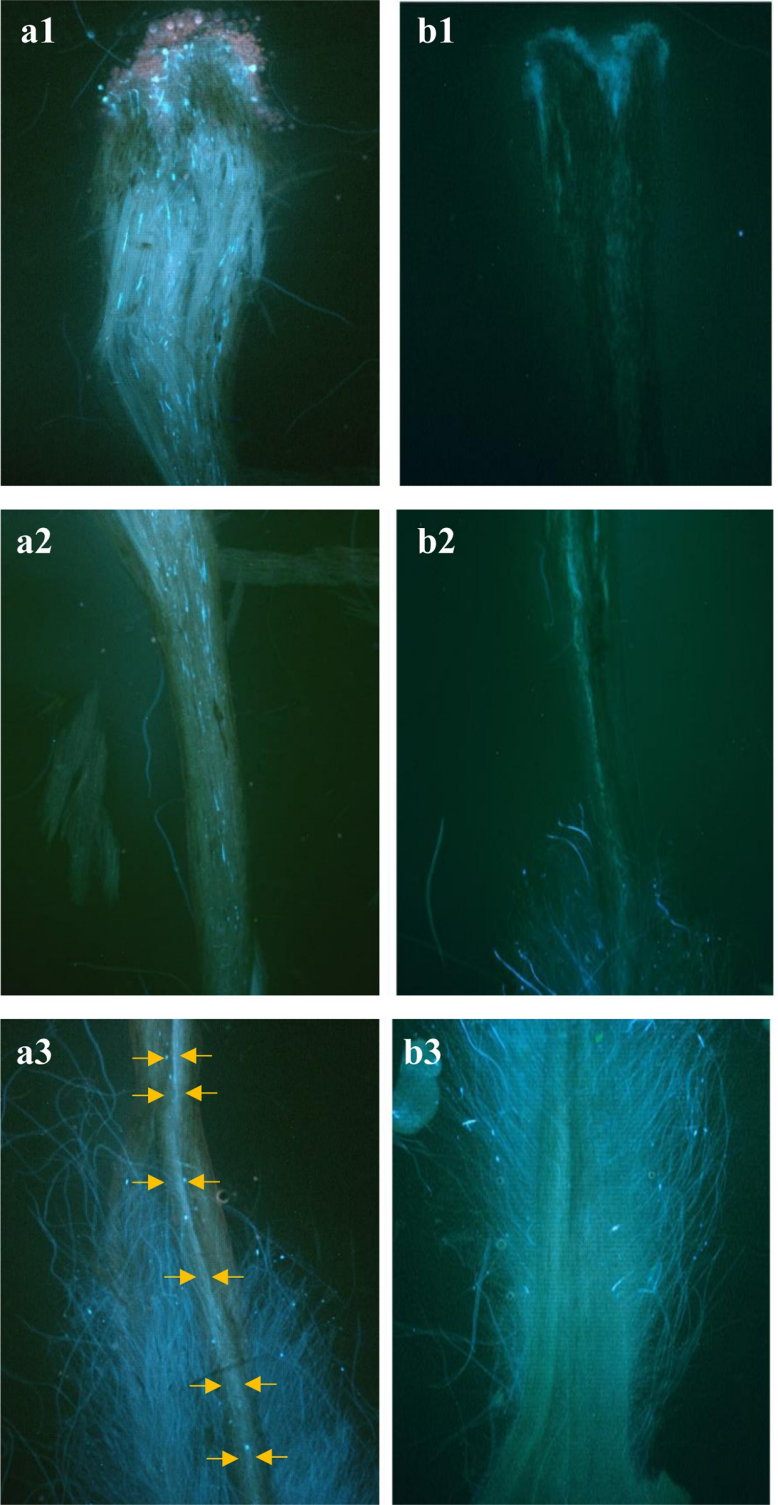
Pollen tube growth after 120 h in two different genotypes of quince: Ardabil2 as a self-compatible (left) and Behta as a self-incompatible (right). a1, b1: one-third at the beginning, a2, b2: one-third in the middle, and a3, b3: one-third at the end. The yellow arrow in a3 indicates the pollen tube.

To evaluate pollen tube growth, fluorescent microscopy was employed to visualize the process in the Ardabil2 and Behta quince genotypes at three different locations along the pistil, including the beginning, midway, and near the ovary (Fig 6). The pollen tube growth was monitored over different time courses in the pistil, with the pollen tube of Ardabil2 reaching at the end of the style after 120 hours of pollination, while no tube was observed in Behta during this time period. Due to the long length of the six images in Fig 6, with each image requiring a full page, we have resized and reduced the length of the images.

Further examination of pollen tube growth using fluorescence microscopy revealed that pollen grains of Behta germinated on the stigma to a limited extent at 120 hours after pollination, with no pollen tubes observed near the ovary (Fig 3 and Table 5). This observation established Behta as a self-incompatible cultivar. Conversely, Ardabil2 pollen grains on the stigma grew normally after 72 and 120 hours of pollination, indicating that Ardabil2 is a self-compatible genotype. Moreover, pollen tubes were observed near the ovary in the crosses of Behta and Ardabil2 after pollination, confirming them as compatible crosses.

**Table 5:** Pollen tube growth during self-pollination and cross-pollination.

Genotype	Time	Beginning	Midway	Near the ovary
Ardabil2♂ × Ardabil2♀	24	*	-	-
	72	*	*	*
	120	*	*	*
Behta♂ × Behta♀	24	-	-	-
	72	-	-	-
	120	*	*	-
Ardabil2♂ × Behta♀	24	*	*	*
	72	*	*	*
	120	*	*	*
Behta♂ × Ardabil2♀	24	*	*	*
	72	*	*	*
	120	*	-	-

In vitro assessments of pollen germination in various quince genotypes revealed typical germination and growth patterns of pollen grains, as shown in Fig 5. To analyze pollen tube growth, fluorescence microscopy was utilized, examining the Ardabil2 and Behta quince genotypes at three distinct positions along the pistil: the start, midpoint, and adjacent to the ovary (Fig 6). Monitoring of pollen tube progression through the pistil over various time intervals showed that the Ardabil2 pollen tube reached the end of the style 120 hours post-pollination, whereas Behta exhibited no pollen tube formation within this duration. Due to the extended length of the six images in Fig 6, necessitating a full page each, their dimensions have been appropriately resized and shortened. Further investigations into pollen tube growth via fluorescence microscopy disclosed that Behta’s pollen grains exhibited limited germination on the stigma at 120 hours following pollination, with no pollen tubes detected near the ovary (Fig 3 and Table 5). This finding identifies Behta as a self-incompatible cultivar. In contrast, Ardabil2’s pollen grains demonstrated normal growth on the stigma after both 72 and 120 hours of pollination, suggesting that Ardabil2 is a self-compatible genotype. Additionally, the observation of pollen tubes near the ovary in cross-pollinations between Behta and Ardabil2 validated their compatibility as crossing partners.

## Discussion

This study adds to our understanding of the genetic diversity using the selected primers and self-incompatibility of quince genotypes, which can be used to develop breeding programs aimed at improving quince cultivars. Interestingly, Talaei et al. [14] found a high level of allelic diversity at the self-incompatibility locus of the *C*. *oblonga*, which may explain the different levels of self-incompatibility observed in its cultivars and genotypes. This observation aligns with the current study’s findings of new S4 and S5 alleles in certain quince genotypes, indicating that genetic variation in quince remains unexplored.

Phylogenetic analysis revealed the presence of five subgroups of S alleles in quince genotypes, with the sub-cluster grouping of S1 and S2 alleles suggesting a closer evolutionary relationship between these two alleles than the other S alleles. This finding is consistent with the complex S-locus structure of Pyrinae species observed by De Franceschi et al. [9], which involves multiple S haplotypes. The involvement of multiple pollen S genes in the GSI system of Pyrinae provides perspectives on the evolution of S-RNase-based GSI within the Rosaceae family.

The identification of conserved regions and SNPs in the S alleles of quince genotypes, with the C2 region displaying the highest degree of nucleotide and amino acid conservation. De Franceschi et al. [9] reported high conservation of the C-terminal part of the S-RNase protein, including the C2 region, across different Pyrinae species. The presence of intra-allelic variation and the identification of two previously unknown S-RNase allele specificities in pear cultivars by Claessen [23] highlights the importance of understanding the molecular basis of self-incompatibility to develop practical applications, such as self-compatible cultivars.

Improving yield in quince cultivars requires identifying appropriate pollinizers. Tatari [24] demonstrated that cross-pollination resulted in the highest percentage of fruit set and yield in the ’Isfahan’ quince cultivar, highlighting the importance of using different cultivars with proper overlap at flowering time to increase fruit set and improve yield in quince cultivars. Akbari Bisheh et al. (2016) also reported similar findings and confirmed the presence of S alleles in the S locus of quince genomes, supporting the current study’s conclusions. Overall, understanding the self-incompatibility mechanism in quince and related species is crucial for improving yield and developing more efficient breeding strategies. The identification of new alleles and the high level of allelic diversity observed in the S gene locus of the *C*. *oblonga* tree highlights the need for further exploration of genetic diversity in quince breeding programs.

In this study, Ardabil2 and Behta were specifically chosen among the genotypes due to their high compatibility and self-incompatibility, respectively. Ardabil2 was chosen due to its remarkably low self-incompatibility, while Behta stood out with the highest self-incompatibility observed among all the genotypes examined. By genotyping of these genotypes, we discovered that Ardabil2 harbored S2S4 alleles, while Behta possessed S1S1 alleles. In the case of the S2S4 genotype of Ardabil2, the presence of both S2 and S4 alleles suggested that it possessed two different versions of the SRK and SCR genes at the S-locus. This genetic diversity within the S-locus allowed for a wider range of recognition and compatibility between pollen and the stigma. The interaction between the S2 and S4 alleles likely resulted in a reduced level of self-incompatibility, making Ardabil2 more compatible with its own pollen. On the other hand, the S1S1 genotype of Behta indicated that it carried identical S1 alleles for both the SRK and SCR genes. This lack of genetic diversity at the S-locus restricted the recognition and acceptance of pollen with matching S1 alleles, leading to a higher degree of self-incompatibility. In other words, Behta was more likely to reject its own pollen due to the presence of identical S1 alleles.

The investigation of pollen-pistil interactions in Ardabile2 and Behta genotypes revealed that Ardabile2 was self-compatible while Behta was self-incompatible. Interestingly, when Ardabile2 and Behta were crossed, normal pollen tube growth was observed, the viability of the pollen. These results suggest that quince can exhibit both self-incompatibility and self-compatibility. This finding is consistent with previous studies by Tatari et al. [24] and Radović et al. [25], which reported that quince can exhibit different levels of self-incompatibility in various cultivars. Radović et al. [25] found that ’Leskovacka’ and ’Vranjska’ were self-compatible cultivars, while the others were self-incompatible. In contrast, the recent study by Radović et al. [26] found that incompatible pollen tubes were present in both self-pollination and open pollination, andthat only ’Leskovacka’ and ’Vranjska’ were self-compatible. These results highlight the importance of carefully selecting pollinizers when establishing new quince orchards to achieve high yields.

In our previous study, we observed variations in fruit set depending on the type of pollination performed between the KVD4 (S4S4) and KVD2 (S1S1) genotypes [7]. Pollination using KVD4 genotype pollen in the KVD2 genotype resulted in a fruit set of 30.28%. However, when cross-pollination was conducted by using KVD2 genotype pollen on the KVD4 genotype, the fruit set decreased to 7.82%. Interestingly, self-pollination of the KVD2 genotype yielded a fruit set of only 3.47%, while self-pollination in the KVD4 genotype did not produce any fruit. Self-incompatibility involves a recognition system between the pollen and pistil, enabling the plant to detect and reject self-pollen, resulting in reduced or no fruit set during self-pollination. S alleles can be involved in (in) compatibility or partial self-compatibility in cultivars and genotypes, and it is likely that the type of S allele present at the S locus can have an impact on the intensity of self-incompatibility in genotypes [27].

The high level of homozygosity observed in the S gene locus in the studied genotypes suggests that self-compatibility is more likely than self-incompatibility in quince trees. This is consistent with the findings of Talaei et al. [14], who observed allelic diversity in the S gene locus of quince, which may explain the different levels of self-incompatibility observed in its cultivars and genotypes. The presence of self-compatibility in quince cultivars may explain their high fertility in northwestern Iran, as observed in the Ardabil 2 cultivar. In terms of practical applications, the study by Tatari [24] found that cross-pollination can increase the percentage of fruit set and improve yield in quince cultivars. The study recommends using different cultivars with proper overlap at flowering time to improve and increase the yield of quince. Radović et al. [25] suggested that care should be taken about the choice of pollenisers when establishing new orchards with self-incompatible quince cultivars.

## Conclusion

The study revealed distinct levels of self-compatibility and self-incompatibility across various quince genotypes. Utilizing microscopic and genotyping methods proved effective in both assessing pollen tube growth and identifying novel S alleles. The discovery of two new S alleles underscores the significance of allelic diversity in influencing the range of self-incompatibility observed in these genotypes. Notably, the preservation of nucleotide and amino acid sequences within the conserved regions of these alleles suggests promising targets for future breeding programs. However, it is essential to conduct further studies to fully comprehend the mechanisms driving the differences in self-compatibility and self-incompatibility among quince cultivars and genotypes. Such research is crucial to harnessing the potential of allelic diversity in the development of new breeding strategies.

## Supporting information

S1 Fig Gel electrophoresis for the quince genotypes. (DOCX)

## References

[1] Soyturk A, Sen F, Uncu AT, Celik I, Uncu AO. De novo assembly and characterization of the first draft genome of quince (*Cydonia oblonga* Mill.). Sci Rep. 2021;11: 3818. 33589687 10.1038/s41598-021-83113-3 PMC7884838

[2] Abdollahi H. A review on history, domestication and germplasm collections of quince (*Cydonia oblonga* Mill.) in the world. Genet Resour Crop Evol. 2019;66: 1041–1058.

[3] FAO. FAOSTAT. Food and Agriculture Organization of the United Nations. FAO Rome, Italy; 2020.

[4] Moradi S, Koushesh Saba M, Mozafari AA, Abdollahi H. Antioxidant bioactive compounds changes in fruit of quince genotypes over cold storage. J Food Sci. 2016;81: H1833–H1839. doi: 10.1111/1750-3841.13359 27273124

[5] Khoshbakht K, Hammer K. Savadkouh (Iran)–an evolutionary centre for fruit trees and shrubs. Genet Resour Crop Evol. 2006;53: 641–651.

[6] Baxitovich SR. Suitable for cultivation in karakalpakstan conditions selection of quince varieties. Ta’lim Va Rivojlanish Tahlili Onlayn Ilmiy Jurnali. 2023;3: 10–12.

[7] Tatari M, Abdollahi H, Mousavi A. Effect of pollination on dropping of flowers and fruits in new quince (*Cydonia oblonga* Mill.) cultivar and promising genotypes. Sci Hortic (Amsterdam). 2018;231: 126–132.

[8] Broothaerts W, Keulemans J, Van Nerum I. Self-fertile apple resulting from S-RNase gene silencing. Plant Cell Rep. 2004;22: 497–501. doi: 10.1007/s00299-003-0716-4 14564475

[9] De Franceschi P, Dondini L, Sanzol J. Molecular bases and evolutionary dynamics of self-incompatibility in the Pyrinae (Rosaceae). J Exp Bot. 2012;63: 4015–4032. doi: 10.1093/jxb/ers108 22563122

[10] Nikzad Gharehaghaji A, Arzani K, Abdollahi H, Shojaeiyan A, Dondini L, De Franceschi P. Genomic characterization of self-incompatibility ribonucleases in the Central Asian pear germplasm and introgression of new alleles from other species of the genus Pyrus. Tree Genet Genomes. 2014;10: 411–428.

[11] Chen W, Wan H, Liu F, Du H, Zhang C, Fan W, et al. Rapid evolution of T2/S-RNase genes in Fragaria linked to multiple transitions from self-incompatibility to self-compatibility. Plant Divers. 2023;45: 219–228. doi: 10.1016/j.pld.2022.04.003 37069931 PMC10105083

[12] Vieira J, Pimenta J, Gomes A, Laia J, Rocha S, Heitzler P, et al. The identification of the Rosa S-locus and implications on the evolution of the Rosaceae gametophytic self-incompatibility systems. Sci Rep. 2021;11: 3710. doi: 10.1038/s41598-021-83243-8 33580108 PMC7881130

[13] Kao T, Tsukamoto T. The molecular and genetic bases of S-RNase-based self-incompatibility. Plant Cell. 2004;16: S72–S83. doi: 10.1105/tpc.016154 15010517 PMC2643390

[14] Talaei Z, Abdolahi H, Sorkhi B, Tatari M, Torkashvand M. Evaluation of Diversity and Isolation of the First Self-Incompatibility Allele in Indigenous Quince (*Cydonia oblonga* Mill.) Genotypes of Iran. 2020.

[15] Ortega E, Dicenta F. Suitability of four different methods to identify self-compatible seedlings in an almond breeding programme. J Hortic Sci Biotechnol. 2004;79: 747–753.

[16] Doyle JJ, Doyle JL. A rapid DNA isolation procedure for small quantities of fresh leaf tissue. 1987.

[17] Sanjari S, Shobbar ZS, Ebrahimi M, Hasanloo T, Sadat-Noori S-A, Tirnaz S. Chalcone synthase genes from milk thistle (*Silybum marianum*): isolation and expression analysis. J Genet. 2015;94: 611–617. 26690515 10.1007/s12041-015-0560-7

[18] Yazdani B, Sanjari S, Asghari-Zakaria R, Ghanegolmohammadi F, Pourabed E, Shahbazi M, et al. Revision of the barley WRKY gene family phylogeny and expression analysis of the candidate genes in response to drought. Biol Plant. 2020;64: 9–19.

[19] Mota M, Tavares L, Oliveira CM. Identification of S-alleles in pear (*Pyrus communis* L.) cv.‘Rocha’and other European cultivars. Sci Hortic (Amsterdam). 2007;113: 13–19.

[20] Babaei F, Abdollahi H, Hajmansoor S. Identification of self-incompatibility alleles in some Iranian native pear cultivar. Seed Plant Improv J. 2012;28.

[21] Tamura K, Stecher G, Kumar S. MEGA11: molecular evolutionary genetics analysis version 11. Mol Biol Evol. 2021;38: 3022–3027. doi: 10.1093/molbev/msab120 33892491 PMC8233496

[22] Dalkiliç Z, Mestav HO. In vitro pollen quantity, viability and germination tests in quince. African J Biotechnol. 2011;10: 16516–16520.

[23] Claessen H. Self-incompatibility and the use of gibberellins in pear fruit production (*Pyrus communis* L.). 2021.

[24] Tatari M. Effect of Self-and Cross-Pollination on Fruit Set and other Characteristics of’Isfahan’Quince Cultivar. J Agric Sci. 2023;25: 4.

[25] Radović A, Cerović R, Milatović D, Nikolić D. Pollen tube growth and fruit set in quince (*Cydonia oblonga* Mill.). Spanish J Agric Res. 2020;18.

[26] Radović A., Nikolić D., Milatović D., Radović I., Zejak D., Spalevic V., et al. Incompatible pollen tubes in the quince style and their impact on fertilization success. Not Bot Horti Agrobot Cluj-Napoca. 2023;51: 13083.

[27] Bisheh HA, Abdollahi H, Torkashvand M, Ghasemi A. Pollen Self-incompatibility and Determination of Appropriate Pollinizer for Quince (*Cydonia oblonga* Mill.) Cultivar Isfahan. Seed Plant J. 2016;32: 13–26.

