# Supplementary figures and images for: Identification of self-incompatibility alleles in Quince (Cydonia oblonga Mill.)
Source: PLoS One. 2024 Feb 8;19(2):e0297595. doi: 10.1371/journal.pone.0297595 (PMC10852308; doi:10.1371/journal.pone.0297595)

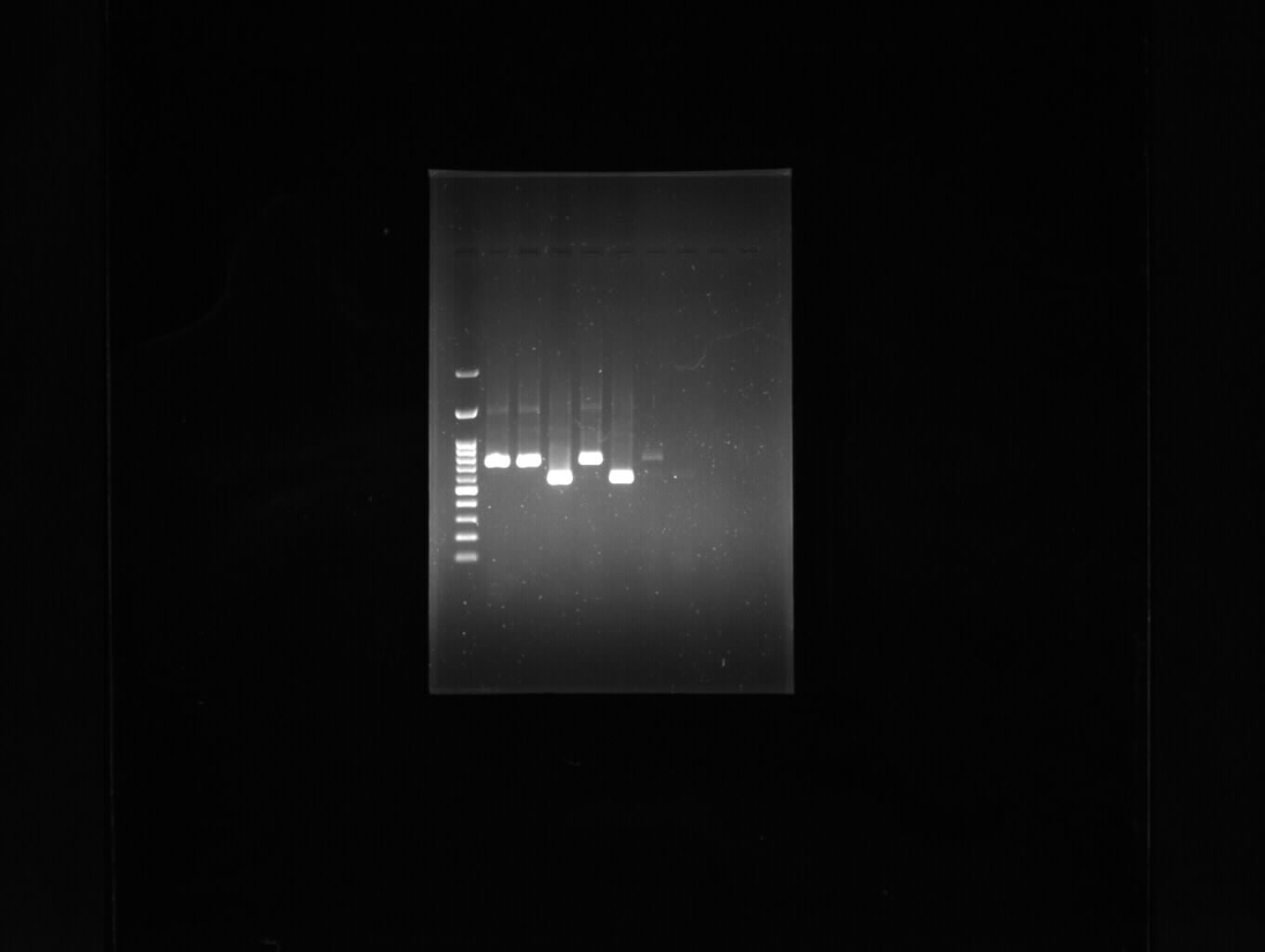


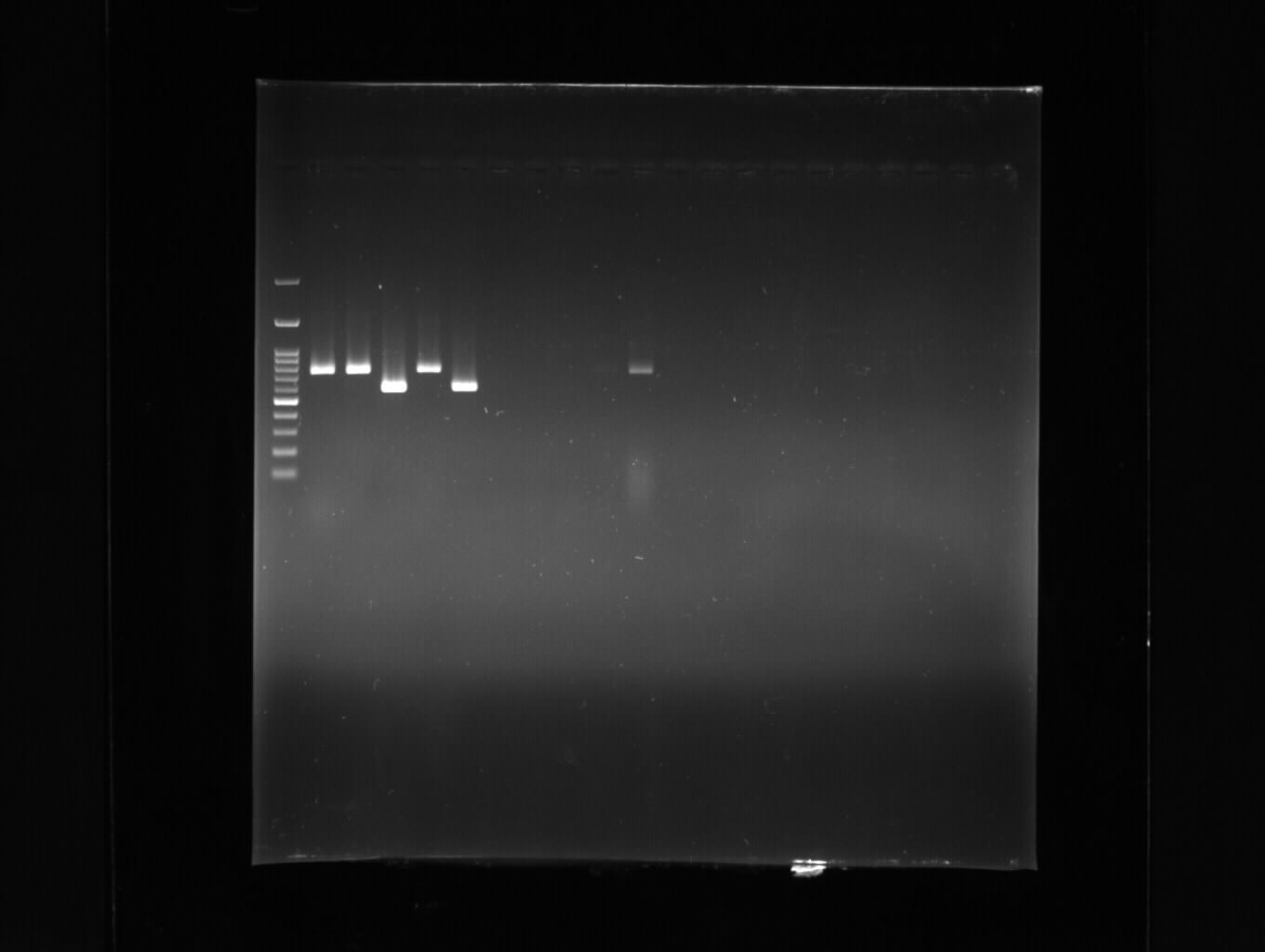


**
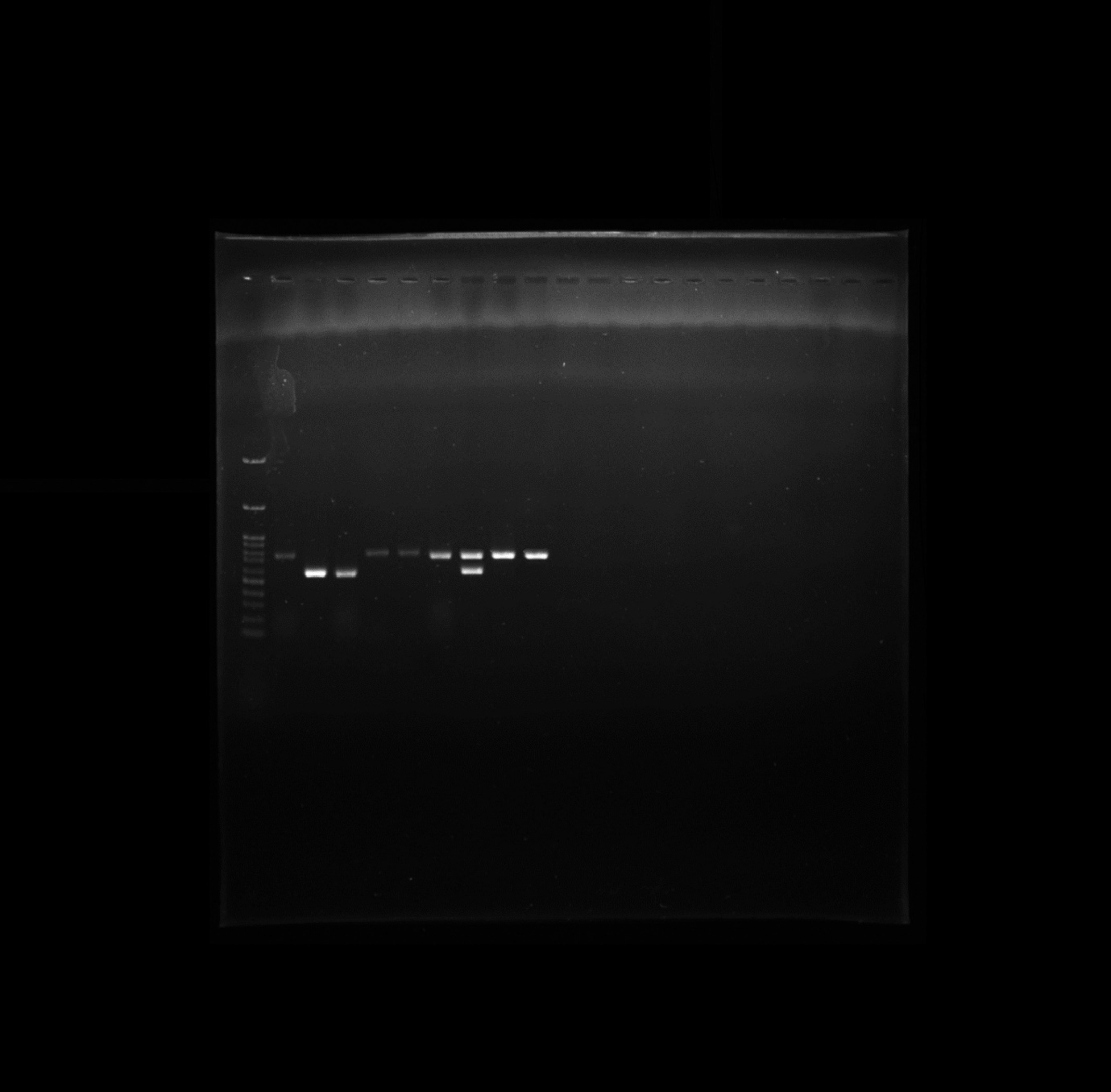
**

**Fig. S1.** Gel electrophoresis for the rest quince genotypes.

Supplement: S1 Fig — (DOCX) [file pone.0297595.s001.docx]
